# *Cannabis sativa*: From Therapeutic Uses to Micropropagation and Beyond

**DOI:** 10.3390/plants10102078

**Published:** 2021-09-30

**Authors:** Tristan K. Adams, Nqobile A. Masondo, Pholoso Malatsi, Nokwanda P. Makunga

**Affiliations:** 1 Department of Botany and Zoology, Private Bag X1, Stellenbosch University, Matieland 7600, South Africa; 20246773@sun.ac.za (T.K.A.); masondo@sun.ac.za (N.A.M.); 2 Cannsun Medicinals (Pty.) Ltd., Cape Farms, Atlantis, Cape Town 7349, South Africa; pholoso@cannsun.com

**Keywords:** cannabinoids, in vitro organogenesis, medical marijuana, plant growth regulators, plant tissue culture, tetrahydrocannabinol

## Abstract

The development of a protocol for the large-scale production of *Cannabis* and its variants with little to no somaclonal variation or disease for pharmaceutical and for other industrial use has been an emerging area of research. A limited number of protocols have been developed around the world, obtained through a detailed literature search using web-based database searches, e.g., Scopus, Web of Science (WoS) and Google Scholar. This article reviews the advances made in relation to *Cannabis* tissue culture and micropropagation, such as explant choice and decontamination of explants, direct and indirect organogenesis, rooting, acclimatisation and a few aspects of genetic engineering. Since *Cannabis* micropropagation systems are fairly new fields, combinations of plant growth regulator experiments are needed to gain insight into the development of direct and indirect organogenesis protocols that are able to undergo the acclimation stage and maintain healthy plants desirable to the *Cannabis* industry. A post-culture analysis of *Cannabis* phytochemistry after the acclimatisation stage is lacking in a majority of the reviewed studies, and for in vitro propagation protocols to be accepted by the pharmaceutical industries, phytochemical and possibly pharmacological research need to be undertaken in order to ascertain the integrity of the generated plant material. It is rather difficult to obtain industrially acceptable micropropagation regimes as recalcitrance to the regeneration of in vitro cultured plants remains a major concern and this impedes progress in the application of genetic modification technologies and gene editing tools to be used routinely for the improvement of *Cannabis* genotypes that are used in various industries globally. In the future, with more reliable plant tissue culture-based propagation that generates true-to-type plants that have known genetic and metabolomic integrity, the use of genetic engineering systems including “omics” technologies such as next-generation sequencing and fast-evolving gene editing tools could be implemented to speed up the identification of novel genes and mechanisms involved in the biosynthesis of *Cannabis* phytochemicals for large-scale production.

## 1. Introduction

*Cannabis sativa* L. (Hemp) (Cannabaceae) is a fast-growing herbaceous species that originated from Central Asia [1,2,3]. The plant has been domesticated for over 5000 years due to its multi-purpose applications. The species is widely utilised as a source of fibre (such as fabrics, ropes, and paper), food, oil, and medicines plus it has a reputation as being used in religious ceremonies and/or for recreational purposes [1,2,4]. *Cannabis* is well known for its hallucinogenic effect and has been widely used to treat a variety of ailments including anxiety, depression, insomnia, convulsive disorders, pain, nausea, asthma, diarrhoea, epilepsy, and malaria; further, it has been used as an aphrodisiac, appetite stimulant, etc. [5,6,7]. Traditionally, the plant is prepared as a decoction or tincture. However, there are many diverse ways in which it can be administered nowadays apart from smoking and vaporising [7]. For example, it can be ingested as edibles (*cannabis*-infused food, drinks, and candies) and applied as oromucosal/sublingual (strips, sprays, and lozenges), transdermal topicals (*cannabis*-infused lotions, balms, and oils) and suppositories [8]. The therapeutic benefits of *Cannabis* and some of its diverse chemical compounds have been pharmacologically documented to treat ailments related to the central nervous system, the neuromuscular system, the respiratory system, the immune system and the cardiovascular effect [9]. 

The medical value of *Cannabis* has been attributed to the various compounds identified and isolated from the plants, e.g., phytocannabinoids and terpenes [10,11]. Cannabinoids have been documented in various studies and clinical trials for problems associated with pain, inflammation, emesis, appetite, obesity, gastro-intestine, anxiety, depression post-traumatic stress, multiple sclerosis, epilepsy, hepatic, neurological and neurodegenerative disorders as well as Alzheimer’s disease; further, cannabinoids possess antispastic, antineoplastic, anticancer and antiemetic activity [3,5,12,13]. Through immense volumes of *Cannabis sativa* and cannabinoid research, this has led to the development of new drugs such as Nabiximols (trade name Sativex^®^) (multiple sclerosis), Epidiolex^®^ (epilepsy), Dronabinol (MARINOL^®^) and Nabilone (CESAMET™) (nausea and vomiting). As the surge in finding more effective drugs from *Cannabis* intensifies, there is a need to properly characterise plant genotype and phenotype to overcome some of the inconsistencies observed in terpene and cannabinoid composition in different strains [1]. Furthermore, the development of suitable protocols for mass production of uniform material from elite *Cannabis* varieties through biotechnological approaches (e.g., in vitro propagation) has become necessary. For this reason, large-scale in vitro propagation of medicinal plants has become an attractive system to meet the high-quality demands of pharmaceutical companies and conservation of valuable elite stock plants. 

**Figure 1:**
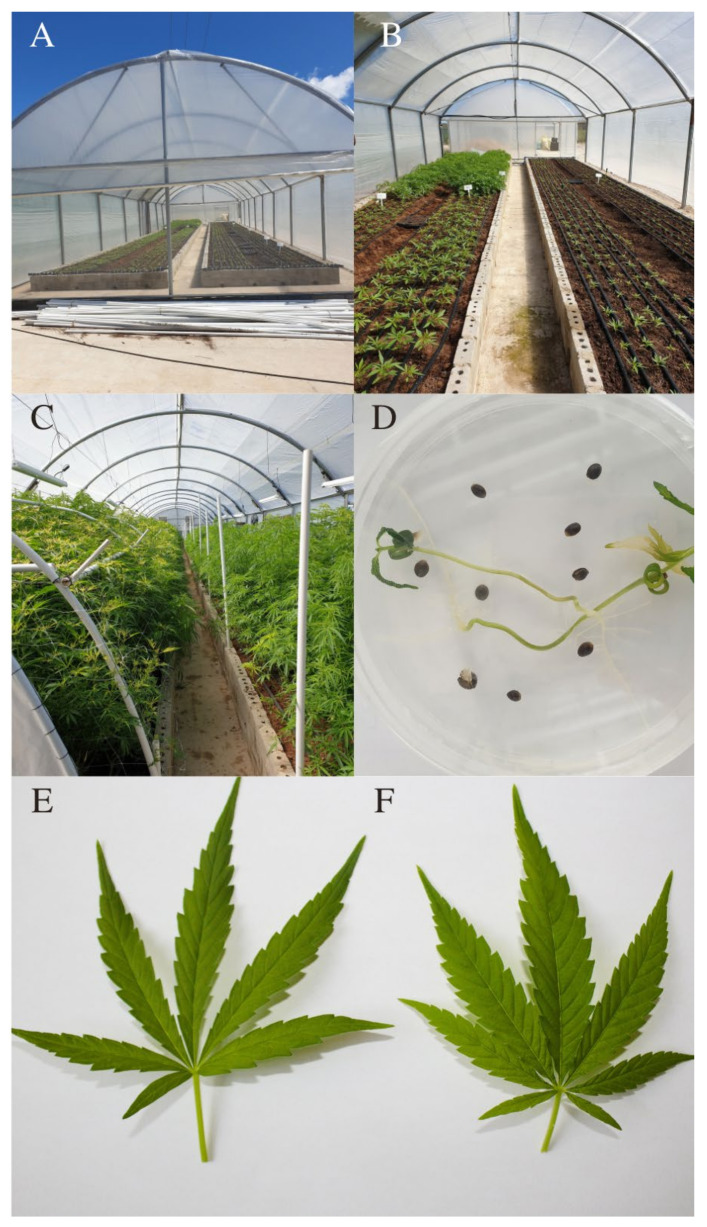
(**A**–**C**) Large-scale conventional cultivation of *Cannabis* leading to tissue culture. (**A**) Large greenhouse for the growth of *Cannabis* at the Cannsun Medhel™ facility in Atlantis. (**B**) Various different growth stages of the Indica variety. (**C**) A 2-month-old mature Lesotho Swazi ready for the flowering stage. (**D**) *Cannabis* seed explants grown on an agar-based medium for tissue culture. *Cannabis* leaf morphology (**E**,**F**): (**E**) *Cannabis* var sativa; (**F**) *Cannabis* var indica.

The shift from conventional propagation (Figure 1A–C) to micropropagation (Figure 1D) allows growers to reproduce disease-free plants in a short period of time that may be identical copies of a specific variant with particularly important desirable phytochemical qualities. The amount of literature that is now available for *Cannabis* micropropagation is continuously expanding and different methods linked to explant use, plant growth regulator regimes, and other microenvironmental conditions in culture are apparent. This paper thus aims to review the already published *Cannabis* micropropagation protocols and their various outcomes. At first, the review briefly gives insights into the industrial/pharmacological uses of *Cannabis*. Thereafter, a comprehensive coverage of the currently available plant tissue culture protocols detailing explant types, plant growth regulator regimes and routes for microplant clonal propagation is presented. As *Cannabis* industries in many parts of the world undergo rapid developments and technological innovations, demands for high agricultural yields in order to derive higher economic value may require high-throughput plant tissue culture procedures that do not sacrifice the quality of the plant as part of the crop production pipeline. The wider implementation of other biotechnologies furthermore is contingent upon the availability of reliable micropropagation procedures that are broadly applicable across a range of *Cannabis* variants.

## 2. General Morphology of *Cannabis sativa*

### 2.1. Plant Morphology (C. sativa Type and C. indica Type)

The *C. sativa* species has three different varieties, namely *C. sativa*, *C. indica* and *C. ruderalis*. The key morphological difference between *C. sativa* and *C. indica* is found in their leaves (Figure 1E,F)*. Cannabis* var. *sativa* has leaves that are much thinner than *Cannabis* var. *indica* [14]. *Cannabis indica*, on the other hand, has wider leaves that are often dark green with a purple tinge, and when mature they turn dark purple. 

*Cannabis sativa* plants have long branches with the lower branches spreading up to 120 cm from the central stalk [15]. The plants can grow to heights of more than 6 m and buds are longer and thinner but much less densely populated than that of *C. indica*. The buds of *C. sativa* require intense light to swell and thicken, whereas *C. indica* does not have such precise requirements. On the other hand, *C*. *indica* plants are bushier and shorter than *C. sativa* plants. These plants rarely grow over 2.5 m and are covered in short branches with dense bud cover that varies in colour, from purple to dark green, with colder conditions inducing colouration that is more intense. These morphological features are important for distinguishing the variants in a cultivation system and remain important for the generation of plants that are true to type. 

### 2.2. Plant Parts Producing Cannabinoids (C. sativa Type and C. indica Type)

Generally, *Cannabis* male and female flowers develop on separate plants but sometimes display a hermaphrodite phenotype [15]. To produce cannabinoids, female plants are favoured over male plants for several reasons including the fact that they produce higher amounts of cannabinoids. The female inflorescence surface has an abundance of glandular trichomes where terpene-rich resins are synthesised. These trichomes can be highly variable in their morphological appearance and *Cannabis* presents with different trichome types, whether they be sessible, bulbous or stalked. Trichome development is regarded by some as being an important indicator for the metabolic maturity of the plant. 

*Cannabis* can be categorised into various different groups/varieties based qualitatively and quantitatively on their chemical profile content, with the ratio of cannabidiol and tetrahydrocannabinol in their leaves used as a general marker for classification of these varieties [16]. The strains are typically distinguished by the chemical composition differences in the resin [1]. *Cannabis sativa* contains high THC and low to no CBD, while *C*. *indica* contains moderate THC and CBD content, with less intoxicating potential as compared to *C*. *sativa* [14]. 

### 2.3. Chemical and Biosynthesis of Cannabis sativa Constituents 

Phytochemical constituents in *Cannabis* are very complex, representing different chemical classes of primary metabolites such as amino acids, fatty acids, and steroids as well as secondary metabolites such as cannabinoids, flavonoids, stilbenoids, terpenoids, alkaloids, and lignans. The main cannabinoid compounds in *Cannabis* include ∆^9^-tetrahydrocannabinol acid (∆^9^-THCA), cannabidiol acid (CBDA), cannabinol acid (CBNA), cannabigerol acid (CBGA), cannabichromene acid (CBCA), cannabinodiol acid (CBNDA) and other minor compounds [17]. Cannabinoid precursors are synthesised from the polyketide pathway and the deoxyxylulose phosphate/methylerythritol phosphate (DOXP/MEP) pathway [13,17,18]. Δ^9^-Tetrahydrocannabinolic acid and CBDA are the main cannabinoids produced for recreational and medicinal use [19]. 

Terpene composition is regarded as the one chemical phenotypic trait that displays large variation across the different strains of *Cannabis* [1]. Most of the terpenes that can be found in *Cannabis* are hydrocarbons. These hydrocarbons are the direct product of terpene synthase (TPS) enzymes [1]. The terpenes found in *Cannabis* resin are manufactured via the isoprenoid biosynthetic route originating in the MEP pathway in the plastids as well as the mevalonic acid pathway [2,20,21].

### 2.4. Therapeutic Uses of Cannabis sativa

Biological properties of cannabinoids are largely attributed to the abundance and localisation of cannabinoid receptors in different organs and tissues. These compounds have proven therapeutic effect against pain, depression, anxiety, arthritis, musculoskeletal diseases, etc. and also have anticancer, antiemetic, antiglaucoma and psychotic properties [12,22]. Experimental studies have shown that activation of cannabinoid receptors, triggered by cannabinoids, results in antitumourgenic activity in most cases that specifically inhibits tumour cell proliferation and/or blocks tumour invasion/metastasis [23,24,25,26]. In addition, cannabinoids are capable of inducing cell apoptosis [22]. Therefore, cannabinoids act as potent anticancer agents against various cancer cell lines such as lymphomas, gliomas, lung cancer, thyroid epithelioma, breast cancer colon cancers and prostate cancers [27,28,29,30,31,32,33]. *Cannabis* formulations or combination extracts have been considered by many as more effective than the use of individual cannabinoids, with the reason behind this being the “entourage effect” [34,35]. This entourage effect is thus defined as a mechanism by which non-cannabinoid compounds such as flavonoids provide synergistic effects when combined with the main cannabinoids, in particular CBD and THC. With more information on the pharmaceutical actions of the phytochemicals of *Cannabis* coming to light, this is fuelling the development of a diverse range of commercialised products linked to its medicinal effects.

As the popularity of *Cannabis*-based products in different countries where the legislation is no longer prohibitive drives global market demands, the commodification and consumption of both medical marijuana and hemp were estimated at $344 billion USD for both the legal and illegal trade and an unprecedented rise is projected in 2019 [36]. Consumers of *Cannabis* were recorded at 263 million in 2018 [37], and with the community of stakeholders and changing social and cultural perceptions towards the use of *Cannabis*-based products being on the rise, even more widespread use of medical marijuana is anticipated in the future. New *Cannabis*-based sectors will emerge, perpetuating the establishment of start-up industries that will continue to demand plant materials at high volumes as lucrative opportunities for market share holders, currently projected to reach $3.6 billion USD by 2027 for legalised *Cannabis* products. The medical sector is thus projected to continue to lead emerging revenue streams as close to 1.2 billion people are currently suffering from medical conditions that could benefit from *Cannabis*-based therapies (https://www.grandviewresearch.com/press-release/global-legal-marijuana-market, accessed on 17 September 2021). Such projections are not only spurring on pharmacological scientific activities, but biotechnological research has also seen a significant rise as a response to future demands for innovative *Cannabis* products. Below, we mainly summarise biotechnological studies that use plant tissue culture as a propagation system for *Cannabis* that may meet the agronomic production challenge of generating mass quantities for harvest. This review also briefly focuses on presently available methods associated with cryopreservation, synthetic seed generation and genetic engineering strategies that have been applied to *Cannabis* for exploitation in industries that are manufacturing phytotherapeutics using various medical *Cannabis* strains and hemp-based products, which are not necessarily consumed for health purposes but are also earmarked to produce fibres for clothes and ropes, wood manufacturing utensils, and important for the production of various commercially important solutions for the cosmeceutics and aligned industries. 

## 3. Methodology 

**Figure 2:**
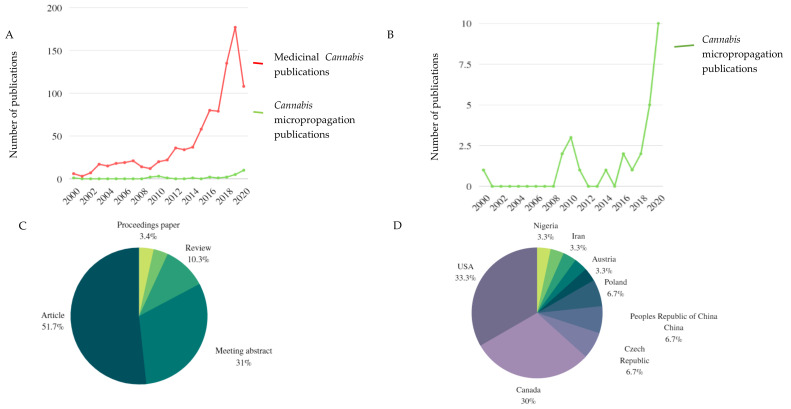
Analysis of *Cannabis* literature (Web of Science). (**A**,**B**) Analysis of *Cannabis* literature in terms of years and number of publications published. (**C**) Pie chart showing an analysis of *Cannabis* micropropagation literature in terms of publication type and percentage of publications published. (**D**) Analysis of *Cannabis* micropropagation literature in terms of country and percentage of overall of publications published.

A detailed literature search using online resources such as Scopus, Web of Science (WoS) and Google Scholar was employed to access scientific studies to construct this review. Keywords such as ‘*Cannabis* micropropagation’, ‘*Cannabis* tissue culture’, ‘*Cannabis* in vitro’, ‘plant micropropagation’, and ‘Medicinal *Cannabis*’ were used in the above-mentioned search engines to generate Table 1, Table 2 and Table 3 and Figure 2. A bibliometric study depicting Figure 2A,B was conducted using WoS (accessed on 25/08/2020) with ‘*Cannabis* micropropagation’ and ‘Medicinal *Cannabis*’ as keywords. Publications from the years 2000–2020 were used to generate a line graph representing the number of Medicinal *Cannabis* and *Cannabis* micropropagation papers published over the last 20 years. The keyword ‘*Cannabis* micropropagation’ was further used to generate literature analysis data for Figure 2C,D. These data were used to generate graphics displaying the number of *Cannabis* micropropagation papers published per country and the number of *Cannabis* micropropagation papers published per paper type including conference proceedings papers, reviews, meeting abstracts, and peer-reviewed articles.

## 4. Legalisation and Propagation Strategies of *Cannabis sativa*

The global ban of *C*. *sativa* for medical and recreational use adopted in 1961 (“Single Convention on Narcotic Drugs”) prohibited the production and supply of the species, and the plant was listed under internationally controlled drugs. This was after *Cannabis* utilisation declined in the early 20^th^ century, possibly from the lack of reproducible research trials and standardised plant varieties for medicinal preparation [13]. In recent years, legalisation of medicinal *Cannabis* in some countries has led to increased demand for industrial-scale production with consistent cannabinoid profiles [38]. Conventional propagation is remaining the mostly commonly used technique even though it is quite costly, time consuming and requires large fields for mass production and rooting of cuttings. On the other hand, in vitro propagation increases production turnover rate, reduces growth duration, uses limited space with controlled environmental conditions, maintains plant genotype and is an effective tool used to improve secondary metabolite content in plants. The prohibition or illegalisation of *Cannabis* use limited the scope of research that was able to take place. In the last decade, following the legalisation of *Cannabis* in some countries, medicinal *Cannabis* research has exploded and there has been a significant increase in publications (Figure 2A).

### 4.1. Conventional Propagation

*Cannabis* can be grown from vegetative cuttings or from seed in outdoor and indoor conditions and is an annual species [15]. Outdoor cultivation is limited to one crop per year while indoor cultivation can generate up to three or four crops per year under controlled environmental conditions. The choice of starting material is dependent on the active ingredient composition needed in the final product. It is relatively easy to propagate *Cannabis* but the rate at which the seeds germinate varies [15]. Despite the plants wide range of agro-ecological conditions, environmental factors (e.g., sowing time, irrigation dose, temperature, soil type and nutrient composition) pose detrimental impact on the overall yield, seed quality, seed oil production and cannabinoid content [39,40,41]. Standardised growth conditions and management practices need to be optimised including light, temperature, CO_2_ concentration, irrigation, humidity, nutrients, growth media (soil and hydroponics), pruning and training for the maintenance of high crop yields [42]. 

### 4.2. Micropropagation of Cannabis sativa

As a result of the significant economic implications of drug-type *Cannabis* for health-related industries, it is becoming more critical to develop ways to produce high-quality biomass with consistent secondary metabolite profiles and this particular goal can be achieved in part by micropropagation [35,43]. That being said, the market for legal hemp for CBD production and medical *Cannabis* is rapidly expanding and producers are turning to advanced scientific procedures as an option to lower the costs of production and offer *Cannabis* varieties that are healthy, scalable, and also of high quality [44]. Even though a few hemp cultivars have been shown to regenerate in vitro, *Cannabis* spp. have a reputation for being somewhat resistant to micropropagation, showing high levels of recalcitrance, and this is integrally associated with the genotype being propagated. Of interest, more success has been possible with genotype MX [45]. This feature makes it difficult to find a reproducible protocol that can be used for routine commercial micropropagation across a range of genotypes and chemotypic variants as different explants are not always responsive to existing published methods. In many instances, establishing a fast-growing and highly regenerative set of plant cultures for *Cannabis* is a challenge. In spite of this, micropropagation still has many potential benefits as a technology for various *Cannabis* industries that offer a diverse range of products and is often a prerequisite step that can assist with other downstream biotechnological manipulations where genetic engineering and gene editing techniques are possibly needed for crop improvement. 

Micropropagation involves multiple processes, indirect and direct organogenesis, with the latter being the most reliable system due to its ability to maintain genetic uniformity between progenies [15]. In vitro propagation of *C*. *sativa* has received little to no attention in the past, with increased publications emerging in 2019 and 2020 (Figure 2A–D). 

Most publications have been research articles, with only 10.3% of all publications being review papers (Figure 2C) and the main focus of these reviews is to pinpoint advances linked to pharmacological effects and biochemical profiles of *Cannabis* plants but none of them indicate micropropagation technologies applied in the species. The countries that presently dominate scientific outputs associated with *Cannabis* research are Canada and the USA at 30% and 33.3%, respectively. This may largely reflect the timelines linked to the legalisation of medical marijuana in certain parts of the USA. In 1973, the first state to decriminalise marijuana in the United States was Oregon. Currently, there are 15 states in the USA that have legalised medical and recreational *Cannabis* [46]. Canada, on the other hand, has a large interest in *Cannabis* production as laws that outlawed the use of marijuana products are continuously being revised and revoked. The overall *Cannabis* market in Canada, including both recreational and medicinal products, was expected to produce up to $7 billion in sales in 2020. A revenue of $2 to $4 billion could be generated by sales associated with legal recreational use, while medical *Cannabis* alone was projected to produce $0.7 billion to $1.7 billion in sales. Of the little research performed on *Cannabis* micropropagation (Figure 2D), Nigeria is the only African country that has conducted some work in the area of plant tissue culture application in *Cannabis* and none of this research has taken place in South Africa despite the legalisation of medical marijuana in September 2018 and increasing producers of *Cannabis*-related products in the country. Because *C. sativa* is naturalised in many different regions of the world, it becomes of importance of to test local strains for their commercial potential and subsequently possibility for agricultural production using both conventional and in vitro microplant techniques as establishment in a cultivation setup is strain dependent.

Even so, efficient in vitro propagation protocols have been developed for direct and indirect clonal propagation as well as ex vitro rooting and acclimatisation systems (Table 1, Table 2 and Table 3). Shoot tips, nodal segments and seeds especially are amongst the most used explants for micropropagation of *Cannabis* (Table 1 and Table 2), with the first report of the use of hypocotyls as explants in Galán-Ávila et al. [47]. Piunno et al. [48] reported the first known shoot regeneration protocol from floral tissues in this species. Explant sterilisation typically involve the use of 70–75% ethanol and sodium hypochlorite with a few drops of Tween 20. Culture initiation, shoot induction and rooting were maintained in medium supplemented (MS) with cytokinins (CKs), auxins and other growth-promoting substances [49]. 

#### 4.2.1. Direct Organogenesis of *Cannabis sativa*

Direct organogenesis refers to the process whereby organs are formed directly on the surface of the cultured explant, bypassing the need for a callus phase. Plant regeneration via direct organogenesis involves various steps: initiation of shoot bud; shoot proliferation; shoot elongation and rooting. This type of plant regeneration is preferred over indirect organogenesis as it avoids unwanted somaclonal variation. Direct regeneration makes use of meristematic tissues and various protocols have been established for propagation of *C. sativa* by use of direct organogenesis via axillary buds, shoot tips and cotyledons as explants [50,51,52] (refer to Table 1).

Several tissue culture protocols established for *C. sativa* have demonstrated the efficient use of different plant growth regulators (PGRs) (Table 1). Lata et al. [50] studied various effects that different concentrations of CK [kinetin (KN), thidiazuron (TDZ) and benzyladenine (BA)] have on the proliferation of nodal explants using axillary buds. Of these CKs, the highest rate of shoot induction was observed with TDZ application when concentrations of 0.11 mg/L, were used. Even though using cytokinins alone is adequate for shoot multiplication, some studies suggest that addition of low concentrations of auxin may in some cases be beneficial [53]. This being said, Wang et al. [51] examined the effects of BA, TDZ, and KN on bud formation in shoot tip explants with or without naphthaleneacetic acid (NAA) and reported a high plantlet response in medium supplemented with 0.2 mg/L TDZ and 0.1 mg/L NAA. The frequency of plantlet regeneration had a bud multiplication rate of 3.22 per shoot tip. An efficient protocol for the micropropagation of C. sativa using *meta*-Topolin (*m*T), a novel aromatic cytokinins has been reported [54,55]. Lata et al. [54] developed a one-step protocol promoting shoot formation and root induction. The study reported a 100% shoot induction at 0.49 mg/L *m*T concentration. A protocol by Kodym et al. [56] used industry-based fertiliser (Canna Aqua Vega Fertilizer A + B Set, The Netherlands), rockwool blocks and forced ventilation, without the need for PGRs, sugar or vitamins. From this study, the authors reported a 95% shoot induction and rooting rate while the stock culture could be maintained for 6 months. Medium supplementation with TDZ (0.11 mg/L) resulted in a 100% culture response, with an average of 13 shoots per explant [50].

Another protocol includes a patent by Grace et al. [57] that reports a method of significantly producing pathogen-free plants and also pathogen-free clones that comprise the heating of a progenitor plant to alternating temperatures of approximately 100 and 85 °F before surface sterilisation with bleach solution. This patented protocol exploits the fast regeneration capacity of plant meristems where the excising of meristematic tips and their transfer onto MS culture medium for further culturing lead to high induction rates for organogenesis. Although the number of patented protocols is few for *Cannabis*, another micropropagation system was patented by Hari [58]. This patent provides a regimen for the generation of new *Cannabis* varieties with modified phytochemical and growth profiles. The innovation described therein subjects plant parts to pectinase digestion (1 mg/mL of pectinase in isotonic buffer, incubated for 3 h) to release plant cells. Following this, the cells are then centrifuged and the cells in the form of a pellet are then cultured on MS medium or Gamborg B5 callus culture media. A second patent by Hari [59] presents a method in which the released plant cells are suspended in a mutagenic solution to obtain mutated *Cannabis* cells prior to their growth on culture medium. Although these protocols are innovative, they can become labour intensive and costly when enzymatic steps are required as part of the procedures of recovering explants for tissue culture. This also means that highly skilled personnel are needed in order to generate digested cells that are still viable that will continue to grow without abnormalities in culture. Tissue culture steps that are simple may be easier to setup in an industrial platform designed for mass multiplication of plants. One of the limiting factors in the tissue culture of *Cannabis* variants is associated with use of different strains, making developed protocols more difficult to establish when another cultivar is being micropropagated. This is illustrated by Codesido et al. [60], where organogenesis responses were difficult to predict even when the same medium was being used. Axillary buds are often used in tissue culture due to their highly regenerative nature and survival rates can even be at 100% (Table 1) but the choice of PGR being used must be of high priority. For example, the use of *m*T proved better for plantlet regeneration compared to the addition of NAA and IBA for direct organogenesis [60].

The explant choice can limit tissue culture growth productivity, and, in some cases, the use of shoot tips and nodal segments yields lowers regeneration frequencies, with explants failing to become highly prolific in their growth patterns [61]. In the study by Galán-Ávila et al. [47], the response of the hypocotyl sections was preferred in comparison to cotyledons when the medium was supplemented with ZEA^RIB^ at 2 mg/L. The addition of 0.02 mg/L NAA was similar to the medium containing the cytokinin alone as a frequency of plantlet regeneration was recorded at 66.67%. Hyperhydricity can be problematic in tissue culture, leading to plants that are abnormal in their growth and development and such cultures are generally more difficult to acclimatise [62]. The use of vented jars that allow for a better gaseous exchange is one method to minimise the hyperhydration of cultured microshoots to generate acclimated plants rapidly that are healthy. Somaclonal variation is kept to a minimum and even avoided by direct organogenesis protocols [54]. Many of the protocols presented were the first of their kind. Nodal explants using *m*T, an aromatic natural cytokinin (cytokinin N^6^–(3–hydroxybenzyl) adenine), have been tested for their successful application on in vitro propagation of *Cannabis sativa*, using a one-step protocol promoting shoot formation and root induction in the same medium [54]. This study also reported that 100% of explants placed on a medium with 0.49 mg/L *m*T produced shoots. The first report of the use of hypocotyls as explants was reported in the work of Galán-Ávila [47]. Piunno et al. [48] reported the first known shoot regeneration protocol from floral tissues in this species. 

*meta*-Topolins are often regarded as a better alternative to replace BA in culture. *meta*-Topolins as a supplement in micropropagation of *C*. *sativa* should be explored further following the success of the protocol presented by Lata et al. [54]. Galán-Ávila et al. [47] and Piunno et al. [48] both reported new explant types that have not been studied before, hypocotyl and floral tissues, respectively. Because of the novelty of these protocols, these should be further investigated to assess efficiency. These explants could also be tested in protocols that have already proven to have high efficiency. The use of seeds as explants seems to be a popular choice and can help avoid pests but requires constant attention when growing as they can develop into male plants. Seeds may be the cheapest method, but seeds need to be feminised prior to germination to ensure there are no male plants (https://www.royalqueenseeds.com/blog-how-to-make-feminised-cannabis-seeds-like-the-pros-n1117 (accessed on 15 December 2020)). Using clones serves as a rapid alternative and guarantees plants with desired genetics. The plants grown from seeds take longer and may not produce identical clones, which is a disadvantage when the intention is to generate plants that are similar in their clonal fidelity. 

Temporary immersion of plant cultures using both semi-solid and liquid cultures has the potential to increase plant yields, allowing for ease of scale-up whilst producing microcultures with minimal symptoms of physiological disorders. Various types of temporary immersion systems are available but thus far the RITA® system has been the one tested for *Cannabis* using shoot tip culture and nodal explants (Table 1) and compared to a self-designed jar in the study by Kodym and Leeb [56]. Although both temporary immersion bioreactors showed good plantlet regeneration, the ease of handling of the plant material during subcultures was thus preferred by the authors. It is likely that other innovations for use of a variety of different temporary immersion bioreactors will be tested in future studies. 

A best-practice regimen may be difficult to obtain for plants that are inherently high-producers of phenolics as wounding that is required for explant sectioning during tissue culture leads to increased production of phenolics as part of the wounding response. The manual handling of the plant material during decontamination steps may also cause physical injury that exacerbates phenolic exudation from the explants. The inclusion of activated charcoal is a common practice as it decreases toxic metabolites, substantially preventing the onset and excessive accumulation of phenolic compounds that may lead to unprecedented explant mortality. For *Cannabis*, several authors have used charcoal as a preventative measure to control phenolics in culture so as not to compromise the health of microplants. The studies of Piunno et al. [48], Grulichová and Mendel [63], Lata et al. [54], and Lata et al. [50] illustrate the application of charcoal for this purpose but, concomitant to this, charcoal leads to the adsorption of PGRs, changing the intracellular phytohormone balances of plants, encouraging the establishment of root initials and extension of lateral roots [64].

#### 4.2.2. Indirect Organogenesis of *Cannabis sativa*

Indirect organogenesis involves shoot regeneration from morphogenic callus. Callus-mediated organogenesis depends on various factors including PGRs and explant type. Shoots, roots, and plant formation from callus cultures can be achieved through the manipulation of PGRs in the medium. Young leaves, petioles, internodes and axillary buds were amongst the most commonly used explants for indirect organogenesis in *C. sativa* (Table 2). Callus tissue are made up of various cell types and the formation of meristemoids relies greatly on the culture medium and also the plant growth regulators applied to this medium. Callus formation is generally induced by increasing cytokinin concentration in the medium and decreasing the concentration of auxins in the medium. Ślusarkiewicz-Jarzina et al. [68] supplemented MS medium with various PGRs to induce callus using a concentration range of 1–4 mg/L kinetin, NAA at 0.5–2 mg/L, 2,4-D (2–4 mg/L) or dicamba (DIC) ranging from 2 to 3 mg/L and petiole explants responded with the highest frequency of callus formation recorded at 82.7% when the growth media had 2–3 mg/L DIC. Although the generation of the callus exhibited high rates, the conversion of this callus to microplant propagules was disappointing, with low organogenesis being problematic. Contrary to this, Wielgus et al. [69] generated callus that could be induced from all types of explants, with cotyledon explants showing the highest callus induction efficiency, and stem explants showing the highest plant regeneration rate. DARIA medium containing NAA, kinetin and BA proved to be an efficient regimen for *C. sativa* plant regeneration [55] and was used to generate in vitro growth of *Cannabis sativa* L., resulting in higher callus induction in comparison to prior studies. 

To optimise in vitro callus induction and regeneration of *Cannabis*, Movahedi et al. [70] investigated the efficiency of BA and TDZ included in the medium at 0.1–3 and 0.1–3 mg/L, respectively, on epicotyl and cotyledon explants to generate callus. These two PGRs were used individually or in combination with 0.5 mg/L IBA. This PGR combination generally leads to direct organogenesis but results from this study showed that callus formation was dominant over direct regeneration, contrary to what is usually expected [70]. A rapid protocol for *C. sativa* plantlet production from young leaf tissue was developed by Lata et al. [71]. Culture initiation took place on MS medium + 0.09–0.35 mg/L IAA, 0.1–0.41 mg/L IBA, 0.09–0.37 mg/L NAA, 0.11–0.44 mg/L 2,4-D with 0.22 mg/L TDZ, while shoots were induced on MS medium + 0.11–2.25 mg/L BAP 0.12–2.15 mg/L KN, and 0.11–2.2 mg/L TDZ. Callus formation was achieved in medium supplemented with 0.09 mg/L NAA and 0.22 mg/L TDZ, whereas the highest shoot induction and proliferation were obtained from 0.11 mg/L TDZ. It was, however, shown that the use of TDZ in plant cell culture might not be the best option due to high levels of DNA methylation in callus cultures that potentially lead to somaclonal variation [72]. Such variations are defined by higher levels of polymorphism, affecting clonal fidelity. Callus formation can be very heterogeneous and can vary tremendously between different types of explants, so many studies test various explants to test their success. Raharjo et al. [73], similarly to Lata et al. [71], investigated the response of leaves as explants and compared leaf material to the use of flowers and seedlings. Interestingly, in that study, leaves were productive in callus formation and flowers were highly effective, giving the most callus in culture. The generation of callus from seedlings was regarded as advantageous and valuable for *Cannabis* regeneration studies. 

Page et al. [74] emphasised the influence of different basal media in their ability to encourage the production of callus, hyperhydricity and low rates of microplant production and the importance of exploring other basal media in addition to MS. In that study, of the five genotypes tested, the MS medium led to higher incidences of callus production when combined with TDZ at 0.11 mg/L but a greater leaf canopy was established when this DKW medium was used instead of MS [75]. The in vitro response of the four different genotypes of medicinal *Cannabis* that were similar may thus indicate the potential to obtain a more standardised protocol that can be used to reliably propagate a wide range of *Cannabis* strains in vitro. Many plant tissue culture protocols for *Cannabis* rely on the use of leaf material or seedlings for plantlet regeneration, even though flowers as an explant source have been shown to be beneficial in other plant species that exhibit recalcitrance to shoot organogenesis. The use of a floral reversion strategy that converts florets to vegetative tissues, under a 12 h photoperiod, led to significantly better shoot development rates in comparison to the use of apical and axillary nodal explants [76]. This method of plantlet production in vitro for *Cannabis* offers an innovative and reproducible technique that could easily be adopted for the commercial production of different *Cannabis* cultivars especially when *m*T at 0.24 mg/L is used as the PGR in the medium [76].

The literature on micropropagation of *Cannabis*, whether it be for the botanical drug markets or other industrial sectors, is a growing scientific concern and future endeavours are likely to result in more innovative approaches to support traditional micropropagation schemes that are currently existing [77]. Although much research has been targeting solutions in finding appropriate growth media and PGR combinations that can produce normal and healthy plantlets of *C. sativa* cultivars, a shift in the research foci of biotechnologists is becoming more obvious. For example, there is an increasing body of literature where physical parameters are being evaluated for their effects on micropropagation and this is a change in thinking, as many previous studies that populate the literature were more focused on finding plant growth regulator combinations that could elicit more prolific shoot regeneration in vitro.

Nowadays, the influence of physical conditions that may control organogenetic responses in tissue cultured plants of *Cannabis*, especially those that are associated with light conditions, is becoming more apparent. Ventilation of the culture vessels for micropropagation in *Cannabis* and other plant genera is thought to be important in producing true-to-type plantlets that are can be readily acclimatised [67,78]. The benefits of investigating physical factors are many. Some of these include *Cannabis* plantlets that are better able to maintain photosynthesis, grow in microenvironments with reduced humidity, and perform gaseous exchange more efficiently, thereby closely resembling plants that are grown ex vitro [67]. To investigate the influence of physical factors, Murphy and Adelberg [67] tested the hedging technique, which involves the removal of the apical meristem, over several subcultures using *C. sativa* ‘US Nursery Cherry 1’, and in this study, the medium was used without the inclusion of PGRs to prevent genetic drift over several continuous culture passages. In non-vented culture jars, higher light intensities were important to maintain a vigorously growing and regenerating culture stock but ventilation in vitro, allowing for better gaseous exchange, assisted with the production of healthier plantlets which could easily be acclimated that also had lower plantlet mortalities ex vitro. These authors iterated the strong apical dominance that is exhibited by *Cannabis* in vitro, which ultimately leads to poor multiplication and shoot branching rates. Therefore, decapitating the apical meristematic tissues thus offers a solution which can promote shoot branch formation and multiplication for *Cannabis* and when this is combined with high light intensities (at 120 μmol m^−2^ s^−1^ PPFD), multiplication rates can improve significantly. Cutting off the apical meristem is easily doable during subculture and should thus be taken into great consideration for a species that is associated with poor multiple shoot cluster formation in a plant tissue culture setting.

As reproducibility of published techniques for micropropagation of other genotypes, apart from the MX *Cannabis* variety, is a growing concern, Monthony et al. [45] tested 10 drug-type variants (genotypes GRC, RTG, U22, U31, U37, U38, U42, U61, U82 and U91) in an attempt to replicate the study by Lata et al. [54]. A genotype-specific response for callus production was evident, with many of the tested genotypes showing browning of the callus due to the accumulation of phenolics. Although callus could be generated for the test genotypes using MS supplemented with a combination of 0.22 mg/L TDZ and 0.09 mg/L NAA, the conversion of the callus to regenerated plantlets was deemed difficult as low plantlet regeneration rates were observed across all the tested *Cannabis* varieties in that particular study regimen. This is a major problem for the establishment of reproducible protocols that can be commercially used as new *Cannabis*-bred lines become more available in years to come.

#### 4.2.3. In Vitro and Ex Vitro Rooting of *Cannabis sativa*

At present, several different PGR regimes are available for use as part of rooting steps that are implemented for *Cannabis* cultivars. In the rooting stage, explants are induced to form roots typically by the application of an auxin. Indole-3-butyric acid (IBA) and indole-3-acetic acid (IAA) are amongst the most used PGRs for root induction. High rooting frequencies were obtained by combining a synthetic auxin such as NAA with a natural auxin such as IAA (Table 1 and Table 2) [55]. *meta*-Topolins have been reported to stimulate in vitro rooting in some plant species without the need for auxin application—in turn, these conditions improve survival rates in acclimatised plants [79]. Some of the methods that are commonly used include a study performed by Wrobel et al. [61] in which they compared the effects of MS medium supplemented with or without different concentrations (0.25–0.75 mg/L) of either IBA or IAA. However, this study showed no significant difference between these two auxins in terms of the rooting rates of microplants. Contrary to the use of plant growth regulators, shoot tip cuttings that are placed directly into rockwool and into a growth chamber promote rooting and rooting rates of 97.5% of the in vitro shoot tip cuttings allow for successfully acclimatised plants within 3 weeks [56,61]. Lata et al. [54] showed that the best concentration for rooting was 0.05 mg/L *mT* and that *mT* concentrations higher than 0.97 mg/L were inhibitory to rooting. Grulichová and Mendel [63] used the same rooting medium as used by Lata et al. [50], whereby full-strength MS medium was supplemented with IBA and activated charcoal. These authors indicated that the highest number of roots were present on plants cultured on media without any PGRs present or on a medium containing 0.5 mg/L *mT*. Lata et al. [50] recorded a 94% response of cultures rooted in a medium supplemented with 0.51 mg/L IBA, with an average of 4.8 roots per explant and confirmed that activated charcoal is effective in root induction.

Plantlets grown in medium supplemented with *m*T successfully rooted well in vitro [54]. Ślusarkiewicz-Jarzina et al. [68] transferred plantlets to a rooting medium comprising of MS basal medium supplemented with 1.0 mg/L IAA + 1.0 mg/L NAA, and 69.9% of plantlets formed roots. To induce rooting, Parsons et al. [65] tested NAA and IBA in four concentrations (0.1, 0.2, 0.5 and 1 mg/L) using regenerated shoots. Of the media explored for rhizogenesis, 0.1 mg/L IBA exhibited the highest rooting rate. Lata et al. [71] similarly showed that shoots rooted best in ½ MS medium with 0.51 mg/L IBA. The presence of IBA resulted in a significantly higher rooting percentage (80–96%) than PGRs IAA or NAA.

The rooting of plants in vitro does assist with acclimatisation, often shortening this particular ex vitro growth phase and this is beneficial when plants are targeted for large-scale agricultural production so as to minimise the acclimation steps in the greenhouse prior to field transplantation. 

The quality of the end products and, in commercial production, economic viability is determined by the successful ex vitro acclimatisation of micropropagated plants [80]. Greenhouse and field conditions have significantly lower relative humidity and higher light levels compared to in vitro conditions, that prove to be stressful to micropropagated plants. The benefits of a micropropagation system can, however, only be realised by successfully transferring plantlets from tissue-culture vessels to the ex vitro conditions [81].

Conventional ex vitro acclimatisation and rooting encompass the steady weaning of plantlets from culture conditions towards ambient light and humidity levels. Direct and indirect regenerated *C*. *sativa* plantlets are subjected to ex vitro rooting and acclimatisation (Table 3). The combinations that are currently used include black peat, granulated peat moss and perlite [47], sterilised soil [61], coco natural growth medium and sterile potting mix-fertilome [54], autoclaved organic manure, clay soil and sand [52] and vermiculite and plant ash [51]. Unlike most soils, cocopeat/coco natural growth medium is unfertilised. This then means that when using this type of media for acclimatisation of plants, an external nutrient solution must be applied, and the pH also needs to be controlled and remain close to neutral. Often the growth of *Cannabis* variants is discussed via online blogs, for example, refer to Mr. Grow It (https://www.mrgrowit.com) [82].

Cocopeat is the preferred medium amongst growers due its many benefits such as its airy and eco-friendly structure that allows roots to thrive in an oxygen-rich environment. Soil, however, retains water better than cocopeat and often already has naturally occurring nutrients that are then assimilated from soil to plant using nutrient acquisition strategies that are readily employed by plants. In such cases, this then minimises time spent watering and preparing nutrient solutions by the grower. The downside, however, is that the roots do not receive as much air as they would in cocopeat, and good air flow to the roots is needed for the stronger and more rapid growth of the *Cannabis* plants. This then makes cocopeat the better choice in terms of increasing yield for the purpose of ex vitro rooting.

In most studies, growth room temperature ranges from 22 to 30 °C with 60% relative humidity [47,50,54]. Wrobel et al. [61] reported a 95% survival rate in the growing chamber and a 90% survival rate in field conditions. Plants propagated with *m*T rooted better when transferred to soil than the shoots produced with TDZ, and plantlets showed a 100% survival rate in acclimatised plants [54]. Lata et al. [50] observed new growth after 2 weeks of ex vitro rooting. Plants reached 14–16 cm in height within 6 weeks of transfer and plants showed normal development and no gross morphological variation. Plantlet survival for 3 months after field transfer was also reported by Wang et al. [51] and the rates were at high levels at 99%. 

#### 4.2.4. Commercial Micropropagation of *Cannabis sativa*

Although it has been many years since the first report of *Cannabis* in vitro cell culture, the existing techniques are inconsistent and limited. Over the previous two decades, the most experienced *Cannabis* corporations have perfected tissue culture and micropropagation procedures, according to popular belief. However, because of the competitive advantage granted inside the industry, most advancements in this in vitro field are kept a ‘trade secret’ [44] and are thus not available in the public space as producers are always concerned about having a competitive edge. *Cannabis* micropropagation has been mostly an underground activity with few peer-reviewed studies [43]. This dearth of knowledge regarding in vitro *Cannabis* protocols has restricted the crop’s biotechnological potential [84] as most species are cultivated in conditions optimised for other species with slight alterations, and are not fully optimised for any given use, due to cost and time constraints.

Despite the many challenges that come with in vitro micropropagation, some successful protocols with minimum risk of somaclonal variation in *Cannabis* have been implemented; however, an efficient and robust protocol is yet to be fully developed for many different varieties that are available to the general public, scientists and agricultural sector [43,54,63,70,74]. There is thus much knowledge in terms of agricultural practices and cultivation procedures in growing this species as a crop, within those that operate in the underground *Cannabis* business, that is not publicly available and much of this is associated with the long history of the plant being prohibited for public consumption and its illegal status in many governments throughout the world. These cultivation regimes that are not shared openly but are often perceived as ‘trade secrets’ may offer ground-breaking protocols that will initiate the movement towards successful commercialisation of *Cannabis* micropropagation on a large industrial scale. The core limiting factor when it comes to in vitro propagation is the large amount of capital needed to setup tissue culture laboratories. The erection of large tissue culture production facilities can need multimillion dollar investments, and therefore relies on the improvement of technology and industries to decrease costs and make micropropagation affordable to all growers [44]. 

Many studies that have investigated micropropagation and other biotechnologies of *Cannabis* are not easy to adopt for large-scale industrial application as they require lengthy periods for plantlets production and are plagued by high costs whilst being inefficient as plantlet regenerants are often showing symptoms of plantlet hyperhydration, poor rooting and acclimatisation frequencies [78]. For these reasons, it thus becomes important to further develop protocols that are more amenable to a commercial setup. The recent study by Zarei et al. [78]. provides a route of micropropagation that could easily be adopted with the objective of generating plants en masse, in a commercial tissue culture setting, as rooting capabilities using rockwool improved significantly. Concomitantly to the use of rockwool, the culture jars that allow for better ventilation were critical in the development of microplants that could easily be acclimatised. 

### 4.3. In vitro Germplasm and Conservation

#### 4.3.1. Cryopreservation 

Long-term preservation of many plant species that are of commercial value is suitably enabled by the application of cryopreservation techniques and this strategy has also been widely applied for rare and/or endangered plant taxa to circumvent biodiversity losses. The preservation of plant tissues using ultra-low temperatures has many wide ranging biotechnological applications. To our knowledge, the first report of the use of such technologies for *Cannabis* germplasm conservation dates back to the late 1980s using hemp callus cultures as the preserved material [85]. Although scientific peer-reviewed articles are few, cryopreservation has been successful for some *Cannabis* varieties. In 2019, Uchendu et al. [86] established a protocol for the in vitro conservation using a Vcryoplate droplet-vitrification microplate method which is more beneficial for long term, high-throughput storage of *Cannabis* lines, namely, cultivars MX and V1-20. This particular method offers significant improvements to traditional techniques as it allows for axillary buds to be pretreated with sucrose and DMSO prior to the freezing stages of germplasm. Cold storage of many medicinal plant requires appropriate cryoprotectants to be used in the different phases of preparing the material for cryopreservation. Experimentation to optimise steps during these phases is thus needed to ensure genetic and morphogenetic potential of plant tissues after being cold stored in ultra-low temperatures. 

More recently, *Cannabis* shoot tips have been the explant of choice being explored for cryopreservation as very few techniques are present in the literature for this purpose. In vitro generated shoots of cultivars MX, VI-20, and B-5 using three different cryopreservation solutions—1) 30% glycerol, 15% ethylene glycol, 15% DMSO in liquid MS medium with 0.4 M sucrose; 2) 40% sucrose, 40% glycerol in liquid MS medium [*w/v*]; 3) 0.6 M sucrose, 3.8 M glycerol, and 20% ethylene glycol in liquid MS—were tested by Uchendu et al. [86]. Of these cryoprotectants, the preservation solutions containing a combination of 40% sucrose and 40% glycerol in liquid MS medium were deemed to be the most effective, allowing for better tissue recovery after the cryopreservation treatments. A genotype-dependent effect was noted during this particular study. Even so, the protocol may be useful for the preservation of other genotypes assisting with germplasm conservation of commercially elite *Cannabis* lines. 

#### 4.3.2. Synthetic Seed Technology 

Germplasm preservation has been enabled by encapsulation of plant cells and tissues in artificial seeds and various encapsulation methods are available, allowing for not only the preservation of germplasm as part of conservation plant management but also the national and international exchange of rare, endangered and/or high-value commercial plant genetic resources [87]. Some of the most popular routes for synthetic seed formation include the use of sodium alginate on its own or when combined with potassium alginate, calcium alginate, carrageenan, gelatin or sodium pectate to create an artificial endosperm that is afterwards stored under low temperature conditions. Various tissue types such as shoot buds, axillary buds, shoot tips, somatic embryos or somatic embryogenic callus, cell micro-aggregates, to name a few, allow for encapsulation and subsequent regeneration under in vitro and ex vitro conditions [88]. These artificial seeds are often stored in a cryopreserved state and in recent years, axillary nodal segments, apical shoot buds and stem sections are being favoured for synseed production. 

Artificial seed technologies are highly relevant for plant species whose in vitro regeneration capacities remain unpredictable using conventional micropropagation. The advantages of this technology are many as this allows for better handling of plant material when it is being transported as part of national and international plant resource exchange programs. This is particularly relevant for a medicinal crop such as *Cannabis*, where many legal restrictions prevail in different regions and countries. The low cost in preserving high-quality genotypes that may be rare and/or economically important, at a large scale, is an added benefit [87]. Commercial growers of medicinal *Cannabis* and other hemp products have acknowledged the advantage of using artificial seeds as the synthetic seeds can be transplanted directly after transport into growing medium for a single genetic clone, saving time and manual labour associated with the generation and rooting of cuttings whilst circumventing the self-crossing problem associated with *Cannabis* agriculture [89]. 

For *Cannabis*, the present exploration of this technology is at its infancy and reliable protocols are thus largely lacking. However, axillary buds and nodal segments have been used to produce an artificial seed [90,91], respectively. Axenic shoots encapsulated in a hydrogel matrix of 5% sodium alginate with 50 mM CaCl_2_.2H_2_O proved to be the most viable route successful synseed production [90]. In the study by Lata et al. [90] the inclusion of an antimicrobial agent, Plant Preservative Mixture™, at 5% was beneficial in controlling the onset of microbial contamination, allowing for aseptic post-encapsulation plant regeneration. The genetic fidelity of encapsulated in vitro seeds that regenerated in shoot proliferation MS medium containing TDZ (at 0.11 mg/L) was then tested using a microsatellite study after rooting of these plant propagules in MS medium with 0.51 mg/L IBA as the rooting solution [91]. 

In an effort to optimise in vitro conditions for the production of undifferentiated callus and somatic embryogenic callus, the study by Hesami et al. [92] utilised a computer-generated machine learning algorithm as a visualisation tool. Various concentrations of 2,4-D and kinetin were tested and the embryogenic tissues were best generated with 0.5 mg/L 2,4-D and 0.25 mg/L kinetin or a combination of 1 mg/L 2,4-D and 0.5 mg/L kinetin, albeit at low rates (10 or 20%, respectively). Therefore, the use of machine learning may provide more efficient combinations that could assist with high recovery of embryo-like structures from callus. With the intention to refine the medium components and carbohydrate resources in DKW and MS media, Hesami et al. [93] employed machine learning algorithms to test predicted concentrations of glucose and sucrose for their efficiency in promoting seed germination and seedling development using an in vitro-based assay. These authors tested the accuracy of the concentrations generated by predictive models. When glucose and sucrose were applied at higher concentrations than standard applications, there were no noticeable differences when the data generated by the model were compared to the experimental results. It is thus our view that utilising machine learning techniques can further assist with generating optimal concentrations of ingredients that can be specifically adopted for artificial seed formation. 

The application of synthetic seed technology at the moment is currently hampered by a paucity of available scientifically validated protocols and this is an avenue of research that may see more interest in the future due to its potential in assisting with clonal lines of superior fidelity that may fit well in a commercial pipeline for *Cannabis* cultivation and genetic transformation and editing, in general. 

### 4.4. Genetic Engineering and Gene Editing 

The establishment of micropropagation protocols for a particular species, cultivar or strain often precedes the utilisation of genetic engineering and gene modification technologies as they offer alternative routes to produce interesting metabolites that are of industrial value. With this in mind, several different strategies are available for genetic manipulation despite the plant showing recalcitrance to in vitro regeneration [94] and genetic transformation [95]. *Agrobacterium*-mediated transformation has been explored by Feeney and Punja [94] and the production of hairy root cultures of five different strains (namely, Futura77, Delta-llosa, Delta405, CAN0111 and CAN0221) were established using seedling leaf material, and hypocotyls, and cotyledons to produce both axenic callus and root cultures using the Ti and Ri plasmid systems, respectively [95]. Such cultures are thus amenable to use as scientific tools to study the biosynthesis pathways associated with *Cannabis* and for their application in industrialised production of *Cannabis*-derived metabolites. Experimentation with the *Agrobacterium* co-cultivation strategies to enable for successful production of transgenics is an important consideration [96]. Of the strains tested— A4, ATCC15834, MSU440, and A13 (MAFF-02-10266)—the *A. rhizogenes* MSU440 strain was the most efficient in generating various *rol*-transformed transgene lines. Successful genetic transformation using *Agrobacterium*-mediated technology is often challenged by different responses associated with using a variety of different strains and the study by Sorokin et al. [97] showed positive transient expression when seedling explants were transformed with a strain of *A. tumefaciens* (EHA105) harbouring the pCAMBIA130-*uid*A GUS reporter gene. Although many of these studies aim to ultimately increase metabolite yields in plant systems, other researchers and biotechnology companies intend to focus on using microbial systems for the heterologous manufacture of desired compounds for en masse production of cannabinoids that occur in nature in trace quantities [98]. With this in mind, it becomes critical to better understand the regulatory mechanisms that control specialised metabolism in *Cannabis*. 

The application of CRISPR Cas9 gene editing techniques also holds promise for use in both *C. sativa* and *C. indica* and with future applications being imminent as CRISPR Cas 9-mediated editing has several advantages for *Cannabis* metabolite engineering. As detailed by Deguchi et al. [98], gene knockouts that target multiple branch pathways may alter metabolic flux, leading to increased production of cannabinoids and terpenoids of pharmaceutical interest. Base editing of single-nucleotide polymorphisms associated with the *Cannabis* genome also offers superior methods that will likely have a higher public acceptance than conventional genetic modification strategies for the production of elite chemotypes of *C. sativa* [98]. Such technologies are also highly suitable for molecular breeding for traits not only associated with phytochemical composition of *Cannabis* strains but also to impart better disease and pest resistance, polyploidy manipulation plus the alterations to general patterns of plant and growth development of *Cannabis* plants [83,98]. 

Broadening the scope of using gene manipulation techniques in *Cannabis* has met with some stumbling blocks despite major advancements in determining the regulatory and biosynthesis genes that control specialised metabolic pathways of key medicinal metabolites uniquely produced by *Cannabis* that could be used for pathway engineering [83]. The main obstacle hindering successful gene engineering is associated with the inability to produce stable transgenics that proceed past the transient transgene expression phase as available plantlet regeneration procedures are unfortunately not presently available for many genotypes of *Cannabis*. The most comprehensive study thus far in solving these problems is that of Zhang et al. [83], where the authors tested 100 hundred strains of hemp obtained from the national germplasm bank of Institute of Bast Fiber Crops (IBFC). This was mainly motivated by the fact that genotype-specific transformations for *Cannabis* are limited in their application across many commercial varieties. 

CRISPR-Cas9 mutagenesis and *Agrobacterium*-derived genetic transformation with several genes (*ZmWUS2*, *NbSTM*, *NbIPT*, *OsGRF4* and *AtGIF1*) that promote somatic embryogenesis and act as gene regulators of pathways that control organogenetic plant responses in other model plant species were explored therein. Although the hemp strain referred to as YUNMA7 is a commercial variant that is most important in China was included in the experiments, the best *Cannabis* strain was determined to be the DMG278 type. With the aim of genetic transforming hemp, this was then chosen as the main experimental model as it was more amenable to *Agrobacterium*-mediated genetic transformation using an AGL1 strain. Transgenics produced in this way were ultimately grown as F2-generation transgenic crops in the field. Together with the production of transgenics, gene editing experiments used a protoplast-based method that was tested initially using six different guides for the CsPDS1 target locus. Although the production of viable protoplasts was at times inefficient, a cocktail of enzymes, in particular, 12 g/L pectinase and 5 g/L cellulose R10 enzymes, allowed for a greater pool of protoplasts that were transformed with the pAT-GFP plasmid. The explant choice, transformation with developmental regulators and the use of gene editing tools as a combined set of processes enabled for the regeneration of hemp transgenics to be successful, setting a new paradigm for exploitation in functional genomics research and for using synthetic biology approaches for improving *Cannabis* plants [83]. In another study, a protoplast-transformation technique using in vitro plantlets of the strain Cherry x Otto II, that inherently produces low THC and high levels of CBD, was used as starter material to introduce the DR5:GFP auxin-sensitive reporter gene together with the p35:RFP expression cassette. Flow cytometry analyses showed GFP-linked fluorescence of the heterologous *Arabidopsis* auxin-responsive element, eliciting IAA-related changes in transgenic protoplasts [99]. 

According to Ahmed et al. [100], plant genetic engineering that employs nanoparticle technology presents novel and revolutionary approaches that can overcome challenges associated with plant species that have proven recalcitrance to *Agrobacterium*-based gene modifications. In addition to this, nanoparticle-assisted transformation may utilise passive infiltration of plant tissues, making it easier to introduce multigene constructs into target tissues that would otherwise require sequential transformation steps with *Agrobacterium*. The latter method may thus become labour intensive and be plagued by poor transformation frequencies when the purpose is to introduce multiple genes into target tissues. As a proof of concept, Ahmed et al. [100] showed transient gene expression in *Cannabis* of two soybean transcription *GmMYB29A2* and *GmNAC42, cloned into* the pGWB6 plasmid, that control the synthesis of glyceollin, which functions as a defence metabolite. Lower leaf surfaces of the Tygra variety were passively infiltrated with poly-ethylenimine cationic polymer-modified silicon dioxide-coated gold (PEI-Au@SiO_2_) nanoparticles using a multigene delivery system for the MYB transcription factors that were linked to a GFP expression marker. Attempts to use *Agrobacterium*-mediated transformation are ongoing as researchers try to expand the varieties that are being tested with this method. Concomitantly to assessing the effectiveness of agrobacterial co-cultivations with seedlings of the short-day *C. sativa* agricultural lines (i.e., Ferimon, Felina32, Fedora17, USO31 and Futura71) and the day-neutral FINOLA type with the intention of generating transgenic plants, an in vitro regeneration protocol was assessed when plants were placed on 0.2 mg/L NAA: 4 mg/L TDZ MS medium [101]. Although cotyledons, hypocotyls and apical meristem explants were co-incubated with *Agrobacterium tumefaciens* LBA4404 (pBIN19), as expected, not all these explants had similar transformation rates. Hypocotyls were most amenable to *Agrobacterium*-mediated gene modification, recorded at 53.3%. Some regenerants had an albino phenotype on the 100 mg/L kanamycin selection medium but spontaneous in vitro regeneration and rooting were evident in certain transformed lines. This study by Galán-Ávila et al. [101] also indicated that the Futura75 strain had the highest transformed hypocotyl-derived transgenics that were transiently expressing the GUS reporter gene, whereas for the FINOLA, Fedora17 and Felina52 varieties, no transgenics were produced, further emphasising on the genotype-dependent specificity aspect that is integral to biotechnological manipulations of *Cannabis*. 

The difficulties in obtaining transgenic plants of this plant continue to hinder scientific progress in studying its genes using functional screening assays, making it impossible to gain deep insights into the regulatory mechanisms that control specialised metabolite production in *Cannabis*. Because the unique enzymes that are responsible for cannabinoid synthesis have been elucidated using genome sequencing tools, regulation of this pathway is highly interesting for biotechnologists and natural product researchers alike. Protoplast transformation appears to be a more dependable and viable pathway for greater number of strains. With the intention to use this method for CRISPR/Cas9 gene editing, to study protein–protein interactions and other genetic-biochemical regulatory mechanisms that control cannabinoid metabolism, Matchett-Oates et al. [102], in a recent study, targeted a high-THC-producing *C. sativa* genotype to examine PEG-mediated protoplast transformation from leaf mesophyll cells. The transformation was confirmed to be correlated to the plasmid and PEG concentrations to elicit transient GFP expression. Even though PEG-assisted protoplast transformation was optimised for a high THC-containing strain, two other strains (Cannbio2 and a high CBD strain) responded adequately with protoplasts that could also be used for future gene editing manipulations and other molecular-based scientific investigations. 

It is thus clear that the possibilities that genetic engineering and gene editing present for furthering our general understanding of cannabinoid metabolism and its regulation provide strong incentives to continue researching simple, less labour intensive, viable protocols for producing a broad-spectrum of genetically modified *Cannabis* strains. Such protocols, that can be routinely applied across many different laboratories, are most likely imminent as this research area has gained considerable attention from biotechnologists with a vested interest in turning *Cannabis* into a model species to study its metabolism. 

Non-transgenic methods using chemical mutagens are also suitable for polyploidy generation in *Cannabis* and a tissue culture step is often a prerequisite for the generation of plants with different chromosome composition than the mother stock [103]. As an example, Parsons et al. [65] generated *Cannabis* polyploids with higher CBD content and sesquiterpene accumulation via tissue culture albeit rooting was lowered in the tetraploids developed using oryzalin, a compound that alters microtubule formation during mitosis. Another example is illustrated by the work of Kurtz et al. [103] using colchicine as the chemical mutagen to induce increased number of chromosomes in in vitro-derived lines from germinated seedlings. A tetraploid hemp line was chosen as having the best potential for downstream breeding applications despite the likelihood of inherent genetic variation that may be produced in some seed. 

Female plants are favoured in *Cannabis* farming, with feminised seed being highly sought after. Polyploids are useful for plant breeding as more desired phenological traits and quality assured phytochemical traits may be expressed in bred plant lines [103]—such manipulation is of high relevance for producing elite *Cannabis* variants. 5. Conclusions and Future Prospects

Micropropagation via direct organogenesis or indirect organogenesis may be a useful tool in propagation of *Cannabis sativa* for mass production of the crop yields with high vigour whilst space requirements are minimised for mass amounts of plant clones. Direct organogenesis allows for genetic preservation of the plants, producing genetically identical clones that are vital for industry use. Micropropagation of *Cannabis* is a fairly new field that is, however, rapidly developing with the opening of new industrial markets globally that have been spurred on by the legalisation of *Cannabis* derived products. Although there have been protocols developed, many more trials and combinations of PGRs experiments are needed to gain further insight into the development of an appropriate species-specific micropropagation regimen that elicits highly prolific organogenesis in culture of morphologically normal and healthy plants. The exploration of different strains of *Cannabis* is also still at its infancy and available protocols are not necessarily highly efficient as they are plagued by low regeneration capacity. To reiterate, more strain-specific investigations are thus also urgently needed to provide a better understanding of strain-related differences with respect to in vitro culture. There is thus room to investigate methods that will allow for efficient, reliable and easy-to-reproduce regimes that are characterised by high multiplication rates and rapid ex situ plant establishment under greenhouse and/or field conditions. Many of the micropropagation regimes discussed in this review do not necessarily include a post-culture analysis of the phytochemistry once plants have been acclimated. This is important for quality control purposes if the application of in vitro cultivation techniques is deemed to maintain the chemical integrity of desired commercial chemotypes. Several different approaches may be useful in this regard including microsatellite analyses to determine genetic-associated somaclonal variation resulting from microculture conditions. Epigenetic effects that illicit unprecedented genetic changes have the potential to alter chemical fingerprints of cultivated *Cannabis* and, together with high-throughput metabolite fingerprinting, this may be useful in monitoring such changes as these can become heritable in subsequent generations. This is particularly important as most *Cannabis* is used for an industry dependent on phytochemical consistency. In the future, it is likely that cryopreservation as a germplasm conservation strategy may be applied for long-term preservation of unique chemotype variants of *C. sativa*. The preservation of heirloom seeds using synthetic seed technologies may be complimentary in the augmentation of breeding activities that solely focus on *Cannabis* and its relatives. 

In addition, when the issues associated with difficulties in propagating *Cannabis* in vitro are no longer an impediment to the generation of healthy and true-to-type clonal propagules, many other genetic engineering applications including the use of fast-evolving gene editing tools will come into routine application in many different laboratories and biotechnology start-ups. This will lead to a revolution in the innovations associated with both medicinal marijuana and hemp. 

## Figures and Tables

**Table 1:** In vitro clonal propagation of *Cannabis sativa* via direct organogenesis *.

Explant	Explant/Decontamination	*Steps* and Culture Medium	Experimental Outcome	Pros	Cons	References
Seeds	Seeds: sterilised in 75% (*v/v*) EtOH for 1 min, rinsed in 5% (*v/v*) active NaCl for 15 min	*Culture initiation* PGR-free MS medium	Best explant response (59–70%) and highest number of shoots per explant recorded for shoot tip explants cultured on medium supplemented with TDZ	Did not utilise PGRs with cytokinin activity, which minimised the risk of soma clonal variation	Regeneration was low, 74% of nodal segments and 82% of shoot tips not growing	[61]
	In vitro shoot tips and nodal segments with one axillary bud without leaves (seedlings)	*Shoot induction* MS medium + BAP (0.5–2.0 mg/L), TDZ (0.1–0.5 mg/L), *m*T (0.1–1.0 mg/L)	Best regeneration rate obtained from TDZ at 0.2 mg/L. Nodal segments less responsive and growth of only one shoot per explant regardless of the tested PGR	Shorter micropropagation duration time. Does not require elongation step	TDZ use related to phenotypic vitrification, leaf rolling, leaf narrowing and supressed growth of shoots. High BAP and *m*T concentrations also related to phenotypic changes in regenerated plants	[61]
	In vitro plantlets	*Rooting* ½ MS medium + IAA (0.25–0.75 mg/L) and or IBA (0.25–0.75 mg/L)	No significant difference observed in tested auxins in terms of rooting rates	It can be used for germplasm conservation and breeding. Rooting limited to 21 days due to rapid growth of shoots in culture. Plantlets obtained within 66–70 days	Number of plantlets from single explant was low. Protocol thus not suitable for industrial application	[61]
Seeds	Seeds: surface sterilised in 75% (*v/v*) ethanol for 2 min and 30 s, soaked in NaClO for 25 min	*Culture initiation* ½ MS medium	Hypocotyl was significantly better than cotyledon leaves in terms of shoot organogenic potential	This is the first report of direct in vitro regeneration of plants from hypocotyls	Leaves displayed a poor ability to promote shoot organogenesis	[47]
	In vitro cotyledons, hypocotyls and true leaves	*Shoot induction* Medium + TDZ (0.4–1.0 mg/L), NAA (0.02–0.2 mg/L), BAP (0.5–2.0 mg/L), IBA (0.5 mg/L), 2,4-D (0.1 mg/L), ZEA^RIB^ (1.0–2.0 mg/L), BAP^RIB^ (1.0 mg/L), 4-CPPU (1.0 mg/L)	Medium containing (TDZ 0.4 mg/L + NAA 0.2 mg/L) was the best, achieving the highest shoot induction rate of 22.32%	None	Medium without PGRs and ZEA^RIB^ 1 mg/L + NAA 0.02 mg/L were the worst treatments, without any explant showing response in terms of shoot organogenesis	[47]
Seeds	Seeds: surface sterilised by washing under running water with a few drops of detergent, 0.2% mercury chloride for 13 min	*Culture initiation* MS medium	Plantlets were grown from seeds	None	None	[63]
	In vitro shoot tips	*Shoot induction* MS medium + BAP (0.4 mg/L)/TDZ (0.1 mg/L)/*m*T (0.5 mg/L) + NAA (0.1 mg/L)/IAA (0.1 mg/L)/GA_3_ (2.3 mg/L)	In both varieties, the highest stem was observed when cultured on medium supplemented with TDZ and GA3, and the shortest stem recorded on medium supplemented with TDZ and NAA	None	The presence of NAA strongly influenced callus formation and general shoot architecture. Difficult to tell which extent longer stems are a genotypic trait	[63]
	In vitro plantlets	*Rooting* MS medium + IBA (0.5 mg/L) + activated charcoal	The most vital plantlets of both genotypes with the highest number of roots were observed on medium where phytohormones were not present or on medium supplemented with *m*T (0.5 mg/L)	Culture media supplemented with *m*T without any phytohormones produced the best overall appearance of plantlets	None	[63]
Seeds	Seeds: soaked with H_2_SO_4_ for 20 s, sterilised in 75% ethanol for 2 min, 3% (*v/v*) NaClO for 20 min	*Culture initiation* MS medium	Seeds grew up to seedlings and cotyledons were excised as explants to induce in vitro shoots	None	None	[52]
	Cotyledons excised from seedlings (aseptic seedlings obtained from sterilised seeds)	*Shoot induction* MS medium + TDZ (0.1–0.4 mg/L), BA (4–8 mg/L), ZT (0.5–1.5 mg/L) with or without NAA (0.1–0.6 mg/L)	Cotyledon cultured in medium containing TDZ with or without the addition of NAA were capable of inducing formation of a nodular callus. Induction rate lower when using only TDZ. Peak of 51.7% induction frequency in MS medium + TDZ (0.4 mg/L) + NAA (0.2 mg/L)	Rapid shoot regeneration. No limitation of cultural season due to the use of cotyledons	This regeneration protocol is genotype dependent	[52]
	In vitro shoots	*Rooting* ½ MS Medium with IBA (0.2–2 mg/L)	IBA (0.5–2 mg/L) had 80% root induction	None	None	[52]
Seeds	Seeds: washed for 20 min with 0.1% antiseptic APSA80 liquid detergent, sterilised in 75% (*v/v*) ethanol for 30 s and 0.1% mercuric chloride for 10–15 min	*Culture initiation* ½ MS medium with 10 g/L sucrose and 5.5 g/L agar	Shoot tips were harvested from 20-day-old sterile plantlets	None	None	[51]
	Shoot tips harvested from 20-day-old sterile plantlets (aseptic seedlings obtained from sterilised seeds)	*Regeneration* MS medium + BA (1–5 mg/L), KT (1–5 mg/L), TDZ (1–5 mg/L) with or without NAA (1–5 mg/L)	TDZ (0.2 mg/L) provided the best bud induction, producing an average of 3.22 buds. 0.1 mg/L NAA was optimal concentration for auxiliary bud induction	CKs stimulated shoot formation and stem enlargement in each explant	Type of CK affected plantlet morphology	[51]
	In vitro plantlets	*Rooting* MS medium + IBA (0.1–0.5 mg/L) + NAA (0.05–0.25 mg/L), IAA (0.05–0.25 mg/L)	85% rooting response in IBA (0.1 mg/L) and NAA (0.05 mg/L) explants	None	None	[51]
Axillary buds	Axillary buds: surface disinfected by maintaining them under stirred tap water for 1 h; 30 min immersion in 15% (*v/v*) bleach, stirred solution	*Culture initiation and shoot induction* MS medium with or without vitamins/Formula βH/Formula βA + 0.48 mg/L *m*T or 0.37 mg/L NAA + 0.41 mg/L IBA with or without MS basal salts, Formula βH basal salts, Formula βA basal salts, with or without MS vitamins	100% survival of axillary buds was observed for all cultivars at least under one studied media. Most of the varieties survived and reacted better without the addition of MS vitamins. Use of PGRs was variety dependent: some cultivars responded better to the addition of *m*T instead to NAA+IBA	This study confirmed that the success of in vitro introduction of *C. sativa* is cultivar dependent	Different cultivars of the same species have a completely different response to the same medium	[60]
Nodal segments with axillary buds	Nodal segments containing young axillary buds: sterilised in 2% NaOCl, 0.1% (*v/v*) Tween 20 for 5 min	*Culture initiation* MS medium + activated charcoal	None	None	None	[65]
	In vitro explants	*Shoot induction* MS medium + 0.1 mg/L NAA + 0.4 mg/L kinetin	None	None	None	[65]
	In vitro plantlets	*Rooting* MS medium + 0.1 mg/L NAA + 0.4 mg/L kinetin + 1.0 mg/L IBA	None	None	None	[65]
	Disinfected axillary buds	*Oryzalin treatments* Shoot induction medium + 17.32, 34.62, 51.95 mg/L oryzalin or MS medium + 6.93, 13.85, 20.78 mg/L oryzalin	62.5% to 87.5% survival rate for explants treated with 6.92 mg/L oryzalin	The treatment of axillary buds with oryzalin is an effective method for chromosome doubling	Poor survival rate of explants treated with high oryzalin concentrations with 0% of explants surviving the 51.95 mg/L	[65]
Shoot tips	In vitro shoot tip cuttings	*Maintenance of stock plants in ventilated glass jars* ¼ Rockwool block placed onto glass preservation jars (3 shoot tip cuttings for each block)	The self-built preservation jars were more suited for the culture of *Cannabis* as they provided more head space	The stock cultures could be maintained for at least 6 months. Excellent-quality plantlets	Wilting plants (blocks too dry/humidity too low). Deterioration of plants due to the blocks being too wet	[56]
	In vitro shoot tip cuttings	*Maintenance of stock plants using RITA^®^ system* Nutrient solution (20 mL), Canna Aqua Vega Fertiliser. RITA container with 3 rockwool blocks each (2 shoot tip cuttings in each block), nutrient solution (75 mL), jars connected via tubing to a 1 bar pressure pump	The RITA^®^ system was more practicable in terms of handling because of the wide opening	Relies on industry-based fertiliser, rockwool blocks and forced ventilation. No requirement of growth regulators. No sugar or vitamins required	Stunted plants or yellow leaves (nutrient deficiency)	[56]
	In vitro shoot tips	*Rooting* Glass vessel, 2 rockwool blocks, nutrient solution (20 mL)	97.5% of in vitro shoot tip cuttings were rooted and acclimatised within 3 weeks inside the growth chamber	None	None	[56]
Shoots	Shoots from immature and mature inflorescences: surface sterilised in ethanol for 1 min, followed by 10% v/v bleach for 10 min, washed in sterile water for 50 s	*Culture initiation* MS medium + TDZ (0–2.2 mg/L)	TDZ was shown to be among the most effective PGRs for shoot proliferation and de novo regeneration	First known report of shoot regeneration from floral tissues	None	[48]
	In vitro explants with regenerating shoots	*Shoot regeneration*/*rooting* MS medium + KIN (0.40 mg/L) + NAA (0.10 mg/L) + activated charcoal	Regeneration was occurring from existing meristematic tissue, but this was not specifically determined	First report of shoot regeneration or plant propagation at reproductive phase	Further work needed to refine the protocol	[48]
Nodal segments with axillary buds	Nodal segments containing axiliary buds: disinfected with 0.5% NaOCl for 20 min	*Shoot induction* MS medium + TDZ (0.01–1.10 mg/L) + 500 mg/L activated charcoal	In TDZ, of the different concentrations tested, the highest average number of shoots was obtained in MS + 0.5 µM TDZ	One step protocol for promoting shoot formation and root induction in the same medium	None	[54]
	In vitro explants with regenerating shoots	*Shoot formation*/*Rooting* ½ MS medium + IBA (0.01–1.01 mg/L), *m*T (0.01–1.21 mg/L)	100% of explants exposed to with 0.48 mg/L *m*T produced shoots. Shoot number and shoot length was higher when using *m*T compared to TDZ. The best concentration for rooting was 0.05 mg/L *m*T	High shoot proliferation rate. Proof of the safety of *m*T for large-scale production. 96% of regenerated shoots were able to develop roots	*m*T concentrations higher than 0.97 mg/L were inhibitory to rooting	[54]
Nodal segments with axillary buds	Nodal segments containing auxiliary buds: sterilised using 0.5% NaOCl for 20 min	*Shoot induction* MS medium + BA (0.01–2.03 mg/L), KN (0.01–1.94 mg/L), TDZ (0.01–1.98 mg/L) with or without GA_3_ (2.42 mg/L)	TDZ was the most effective PGR for shoot proliferation. 100% culture response when using TDZ (0.11 mg/L), with an average of 13 shoots per explant	Regeneration of many plants in a short period of time. GA_3_ can act as a replacement for auxins in shoot induction	TDZ concentrations higher than 1.1 mg/L supressed shoot formation	[50]
	In vitro shoots	*Rooting* MS medium + IAA (0.44–0.88 mg/L), IBA (0.51–1.02 mg/L), NAA (0.47–0.93 mg/L) with or without 500 mg/L activated charcoal	94% response of cultures in IBA (0.51 mg/L) with an average of 4.8 roots per explant	Addition of activated charcoal was effective in root induction	Profuse callus formation was observed when using IAA and IBA	[50]
Nodal segments with axillary buds	Apical nodal segments containing axillary bud: sterilised using 0.5% NaOCl for 20 min	*Shoot initiation* MS medium + BA, KN, TDZ (concentrations not mentioned)	Quality and quantity of shoot regenerants in cultures were best with 0.11 mg/L TDZ	None	None	[64]
	Apical nodal segments containing axillary bud: sterilised using 0.5% NaOCl for 20 min	*Rooting* ½ MS medium + activated charcoal + IAA + IBA + NAA (concentrations not mentioned)	Highest percentage of rooting achieved in ½ MS with 500 mg/dm^3^ activated charcoal supplemented with 0.51 mg/L IBA	None	None	[64]
Nodal segments with axillary buds	Nodal segments containing axillary buds: sterilised using 1.67% (C(O)NCl)₂ + Tween 20 for 8 min	*Shoot initiation*: MS + TDZ (0.011– 1.76 mg/L), *m*T (0.012–1.93 mg/L), BAP (1–5 mg/L), IAA (0.1 mg/L)	MS medium + 0.1 mg/L TDZ resulted in the highest regeneration of shoots. Tissue culture responsiveness was genotype dependent	None	Results demonstrated the recalcitrance of *Cannabis* in tissue culture and its poor multiplication rate	[66]
Apical shoot tip	Apical shoot tip+ node	*Shoot initiation:* DKW medium without PGRs	The highest number of harvested shoot tips was found in the 46 µmol/m^2^/s in non-vented vessels	Unlike traditional micropropagation, this method re-uses the same rooted basal stem section of the initial explant over several apical tip removal cycles, resulting in a higher number of shoot tips	None	[67]

Abbreviations: BA/BAP—6-benzylaminopurine, H2SO4—sulphuric acid, IAA—indole-3-acetic acid, IBA—indole-3-butyric acid, KIN—kinetin, MS—Murashige and Skoog, *m*T—*meta*-Topolin, NAA—1-naphthaleneacetic acid, NaCl—sodium chloride, NaOCl—sodium hypochlorite, PGR—plant growth regulator, RITA—temporary immersion system for tissue culture, TDZ—thidiazuron, ZEA—zeatin, and 4-CPPU—forchlorfenuron. * The list in this table may not be completely exhaustive.

**Table 2:** In vitro clonal propagation of *Cannabis sativa* via indirect organogenesis *.

Explant	Explant/Decontamination	*Steps* and Culture Medium	Experimental Outcome	Pros	Cons	References
Seeds	Seeds: Sterilised in 5% Ca (ClO)_2_ for 6, 8 and 15 min	*Culture initiation* MS medium	Best sterilisation time was achieved after 15 min (5% hypochlorite solution)	None	Hemp seeds were highly contaminated	[68]
	In vitro young leaves, petioles, internodes and axillary buds	*Callus induction*/*indirect regeneration* MS medium + KN (1–4 mg/L), NAA (0.5–2 mg/L), 2,4-D (2–4 mg/L), DIC (2–3 mg/L)	Callus was obtained from all explant types. Petiole explants with 2–3 mg/L DIC had the highest frequency of callus formation with 82.7% of explants	Explants derived from plants growing in pots	Low frequency of callus from internodes and axillary buds. Efficiency of plant regeneration is low	[68]
	In vitro regenerated plantlets	*Rooting* MS medium + IAA (1 mg/L) and NAA (1.0 mg/L)	69.95% of the plantlets formed roots	None	Further experiments needed to develop an efficient plant regeneration system	[68]
Seeds	Seeds: sterilised in 70% ethanol for 10 s and in 1% NaClO for 20 min	*Culture initiation* DARIA medium	Explants of cotyledons, stems, and roots were excised from plantlets	None	None	[69]
	In vitro cotyledons, stems, roots	*Callus induction* DARIA medium + KN (1 mg/L) + NAA (0.05 mg/L)	Callus was obtained from all explant types	The highest efficiency of morphogenic callus induction was noticed from cotyledon explants	Callus formed at root explants was incapable of morphogenesis and plant regeneration	[69]
	In vitro explants	*Indirect regeneration* DARIA medium + BA (0.2 mg/L) + NAA (0.03 mg/L)	Stem explants showed the highest regeneration rate percentage and cotyledon explants showed the highest efficiency in callus induction	The use of three media, DARIA ind+, DARIA pro +, and DARIA root +, supplemented with PGRs, enabled regeneration of plants with relatively high efficiency	None	[69]
	In vitro explants	*Rooting* DARIA medium + IAA (2 mg/L)	Rooted plants were transferred to soil	None	None	[69]
Seeds	Seeds: sterilised with 70% ethanol for 30 s, 2% NaOCl for 20 min and 0.05% HgCl_2_ for 5 min	*Culture initiation* MS medium	Seeds produced seedlings for obtaining explants	None	None	[70]
	In vitro cotyledon and epicotyl	*Callus induction* MS medium + BA (0.1–3 mg/L), TDZ (0.1–3 mg/L) with or without IBA 0.5 mg/L	Cotyledon explant showed better response compared to epicotyl explants in terms of the mass and size of the calli produced in various hormonal combination	The first response of explant to callus formation was observed after 11 days. The addition of IBA in various concentrations of BA had positive influence on callus induction	None	[70]
	In vitro calli	*Shoot induction* MS medium + BA (0.1–3 mg/L), TDZ (0.1–3 mg/L) with or without IBA 0.5 mg/L	Epicotyl explants showed better regeneration rate compared to cotyledon. Epicotyl explant callus treated with 2 mg/L BA and 0.5 mg/L IBA showed high shoot regeneration rate	None	None	[70]
	In vitro regenerated shoots	*Rooting* MS medium + NAA (0.1–1 mg/L), IBA (0.1–1 mg/L)	IBA (0.1 mg/L) showed highest rooting rate	None	Burning was observed in the shoots cultured in media supplemented with NAA hormone	[70]
Young leaves	Young leaves: sterilised using 0.5% NaOCl, 15% (*v/v*) bleach	*Culture initiation/callus induction* MS medium + IAA (0.09–0.35 mg/L), IBA (0.1–0.41 mg/L), NAA (0.09–0.37 mg/L), 2,4-D (0.11–0.44 mg/L) with 0.22 mg/LTDZ	Optimum callus growth in 0.09 mg/L NAA + 0.22 mg/L μM TDZ	Rapid protocol for producing plantlets from young leaf tissue	The formation and growth of the callus was affected by the type of PGR and concentration applied	[71]
	In vitro calli	*Shoot induction* MS medium + BAP (0.11–2.25 mg/L), KN (0.12–2.15 mg/L), TDZ (0.11–2.2 mg/L)	Highest shoot induction and proliferation was observed in 0.11 mg/L TDZ	None	None	[71]
	In vitro regenerated shoots	*Rooting* ½ MS medium + IAA (0.09–1.75 mg/L), IBA (0.10–2.03 mg/L), NAA (0.09–1.86 mg/L)	Shoots rooted best in ½ MS medium with 0.51 mg/L IBA. The presence of IBA resulted in significantly higher rooting percentage (80–96%) than IAA or NAA	None	None	[71]
Leaves, flowers, 4-day-old seedlings	Leaves, flowers, and 4-day-old seedlings: washing with detergent, 70% EtOH for 3 min, sterilised distilled water for 10 min, 2% NaClO soak for 20 min	*Culture initiation*/*callus induction* MS medium + mesoinositol (100 mg/L), thiamine diHCl (10 mg/L), pyridoxine HCl (1 mg/L), nicotinic acid (1 mg/L), 2,4-D (1 mg/L), sucrose (30 g/L) and agar (10 g/L)	Flowers gave more callus while the leaves had less callus production	Callus was easily induced in standard medium	Cannabinoids were not produced in *Cannabis* cell cultures	[73]
	In vitro calli	*Suspension cultures* Liquid MS medium after 2 weeks: one part was maintained in the MS medium while the other was maintained in B5 medium (B5 components, 2,4-D 2.0 mg/L, IAA 0.5 mg/L, NAA 0.5 mg/L, K 0.2 mg/L and sucrose 30 g/L)	Shoots from seedlings produced more callus than the stems and no callus was formed on the roots	None	None	[73,76]
Immature embryo hypocotyls, true leaves, cotyledons and hypocotyls	Immature embryo hypocotyls, true leaves, cotyledons and hypocotyls: sterilised using 2% (*v/v*) NaClO for 25 min followed by 75% (*v/v*) EtOH for 5 min	*Culture initiation:* MS+ nicotinic acid (1 mg/L) + pyridoxine-HCl (1 mg/L) + thiamine-HCl (10 mg/L) + myo-inositol (0.1 g/L) + 3% sucrose + phytagel (2.5 g/L) + 2,4-D (1 mg/L) + KIN (0.25 mg/L) + casein (100 mg/L) *Hydrolysate regeneration:* 1/2 strength MS + 1.5% sucrose + phytagel (3.5 g/L) + TDZ (0.5 mg/L) + 6-BA (0.3 mg/L) + NAA (0.2 mg/L) + IAA (0.2 mg/L) *Rooting:* 1/2 strength MS + NAA (0.2 mg/L) + IBA (0.5 mg/L) + ZeaRIB (0.01 mg/L)	Over 20% of the immature embryo hypocotyls developed embryogenic calli within 5 days. Hypocotyls collected 15 days after anthesis produced more calli than those collected earlier or later	None	Genotype dependence of *Cannabis*	[83]
Leaf	Leaf material from in vitro shoots: no sterilisation mentioned	*Culture initiation/callus induction* MS + TDZ (1.0 μM) *Shoot induction* MS + TDZ (0.5 μM)	Callus was effectively induced in all 10 genotypes, yet the subsequent transfer of calli to shoot induction medium failed to initiate shoot organogenesis in any of the tested genotypes. Regeneration of *Cannabis* from somatic tissues is highly genotype specific	None	This method is not suitable for inducing de novo regeneration across different genotypes	[45]

BA/BAP—6-benzylaminopurine, EtOH—ethanol, HCl—hydrochloric acid, H2SO4—sulphuric acid, IAA—indole-3-acetic acid, IBA—indole-3-butyric acid, KIN—kinetin, MS—Murashige and Skoog, *mT—meta*-Topolin, NAA—1-naphthaleneacetic acid, NaCl—sodium chloride, NaOCl—sodium hypochlorite, PGR—plant growth regulator, TDZ—thidiazuron, ZEA—zeatin, and 2,4-D—2,4-dichlorophenoxyacetic acid. * The list in this table may not be completely exhaustive.

**Table 3:** Ex vitro rooting and acclimatisation of *Cannabis sativa* *.

Plantlet Growth Stage	Growth Conditions	Experimental Outcome	References
Direct Organogenesis			
Plantlet (21 days old)	-Pots with sterilised soil -Under a plastic cover -25 ± 1° C (18/6 photoperiod, 60 µmol m^−2^ s^−1^) -Hardened for 2 weeks before transferring to the field	95% survival rate in the growing chamber 90% survival rate in field conditions Plantlets maintained ability to synthesise cannabinoids	[61]
Spontaneously rooted plantlets	-Pots (2 L) with fertilised commercial substrate (black peat, granulated peat moss and perlite) - Regenerants received foliar pulverisation with water -Small plants were covered with plastic vessels and were progressively exposed to the environmental humidity - 22 ± 1 °C - 60% ± 1% relative humidity	After 1 week of progressive exposition of regenerants to the environmental humidity, the process of acclimatisation ended, and hypocotyl-derived plants displayed a vigorous growth Hypocotyl derived plants showed sexual functionality 8 weeks after in vitro explant inoculation	[47]
Plantlet (age not defined)	-Kept under controlled environmental conditions in an indoor cultivation facility -Well rooted plants washed with tap water to remove all traces of medium -Plants pre-incubated in coco natural growth medium for 10 days before transferring in sterile potting mix-fertilome in large pots -25–30 °C -Light, ∼700 µmol m^−2^ s^−1^ with 16 h photoperiod -60% relative humidity	Plants propagated with *m*T rooted better when transferred to soil than the shoots produced with TDZ 100% survival rate in acclimatised plants	[54]
Plantlet (age not defined)	-Kept in a greenhouse -Plantlets with well-developed roots removed from tissue culture vessel and washed under running water -Propagated in plastic cups containing sterilised organic manure, clay soil and sand (1:1:1) -22 °C -Cool white, fluorescent lights (16/8 h photoperiod, 36 µmol m^−2^ s^−1^)	75% of rooted shoots survived after acclimation	[52]
Rooted shoots (age not defined)	-Kept in controlled environmental conditions grown in an indoor cultivation facility -Rooted shoots were carefully taken out of the medium and washed thoroughly running tap water -Plantlets were pre-incubated in coco natural growth medium thermocol cups for 10 days -Cups were covered with polythene bags to maintain humidity and later acclimatised in sterile potting mix-fertilome -A hot air suction fan was attached with approximately 1 m distance between plants -16 h photoperiod -25–30 °C -60% humidity	95% survival of rooted plantlets transferred to soil New growth observed after 2 weeks Plants reached 14–16 cm in height within 6 weeks of transfer Plants showed normal development and no gross morphological variation	[50]
Plantlets	-Rooted shoots were carefully taken out of the medium and washed thoroughly in running tap water followed by washings with 0.2% (w/v) Bavistin1 and tap water -Washed plantlets were transferred to root trainers consisting of 20 cells, each of 200 cm^3^, filled with perlite and 10 mL water -Plantlets were transferred to plastic pots filled with vermiculite and plant ash, grown in a shade-house -After an acclimation period of 2 weeks, the plantlets were able to be transplanted to the field	95% plants acclimatised 99% plantlet survival for 3 months after field transfer	[51]
Plantlets	-Rooted plantlets were placed in Grodan Gro-Smart Tray Insert (Indoor Growing Canada, Montreal, Canada) in the standard tray with transparent dome (Mondi, BC, Canada) with vents. -The plants were fertilised using SF vegetative fertiliser solution. -Rooted plants received photoperiod and light intensity conditions (150 μmol m^−2^ s −1 and 18/6 h light/dark).	Survival rate above 90% Up to 2260 rooted plantlets were produced per 10 m^2^	[78]
**Indirect Organogenesis**			
Rooted shoots	-Cultivated in pots containing equal ratio of perlite and pit moss -To avoid evaporation, the pots were covered with a transparent cover and placed in growth chambers -25 °C -Covers removed after two weeks and plants were transferred into the greenhouse	70% of the seedlings produced in tissue culture conditions survived and showed normal growth	[70]
Plantlets	-Rooted shoots were carefully taken out of the medium and washed thoroughly in running tap water -Plantlets were pre-incubated in coco natural growth medium thermocol cups for 10 days -Growth cups were covered with polythene bags to maintain humidity, kept in a grow room, and later acclimatised in sterile potting mix (fertilome) in large pots -25 °C	95% survival rate in indoor grow room	[71]

*m*T—*meta*-Topolin and TDZ—thidiazuron. * The list in this table may not be completely exhaustive.

## Data Availability

Data are contained within this article.
